# International Veterinary Epilepsy Task Force recommendations for a veterinary epilepsy-specific MRI protocol

**DOI:** 10.1186/s12917-015-0466-x

**Published:** 2015-08-28

**Authors:** Clare Rusbridge, Sam Long, Jelena Jovanovik, Marjorie Milne, Mette Berendt, Sofie F. M. Bhatti, Luisa De Risio, Robyn G. Farqhuar, Andrea Fischer, Kaspar Matiasek, Karen Muñana, Edward E. Patterson, Akos Pakozdy, Jacques Penderis, Simon Platt, Michael Podell, Heidrun Potschka, Veronika M. Stein, Andrea Tipold, Holger A. Volk

**Affiliations:** Fitzpatrick Referrals, Halfway Lane, Eashing, Godalming, GU7 2QQ Surrey UK; School of Veterinary Medicine, Faculty of Health & Medical Sciences, University of Surrey, Guildford, GU2 7TE Surrey UK; University of Melbourne, 250 Princes Highway, Weibee, 3015 VIC Australia; Department of Veterinary and Clinical Sciences, Faculty of Health and Medical Sciences, University of Copenhagen, Frederiksberg C, Denmark; Department of Small Animal Medicine and Clinical Biology, Faculty of Veterinary Medicine, Ghent University, Salisburylaan 133, Merelbeke, 9820 Belgium; Animal Health Trust, Lanwades Park, Kentford, Newmarket, CB8 7UU Suffolk, UK; Fernside Veterinary Centre, 205 Shenley Road, Borehamwood, SG9 0TH Hertfordshire UK; Centre for Clinical Veterinary Medicine, Ludwig-Maximilians-University, Veterinärstr. 13, 80539 Munich, Germany; Section of Clinical & Comparative Neuropathology, Centre for Clinical Veterinary Medicine, Ludwig-Maximilians-University, Veterinärstr. 13, 80539 Munich, Germany; Department of Clinical Sciences, College of Veterinary Medicine, North Carolina State University, 1052 William Moore Drive, Raleigh, NC 27607 USA; University of Minnesota College of Veterinary Medicine, D426 Veterinary Medical Center, 1352 Boyd Avenue, St. Paul, MN 55108 USA; Clinical Unit of Internal Medicine Small Animals, University of Veterinary Medicine, Veterinärplatz 1, 1210 Vienna, Austria; Vet Extra Neurology, Broadleys Veterinary Hospital, Craig Leith Road, Stirling, FK7 7LE Stirlingshire UK; College of Veterinary Medicine, University of Georgia, 501 DW Brooks Drive, Athens, GA 30602 USA; Chicago Veterinary Neurology and Neurosurgery, 3123 N. Clybourn Avenue, Chicago, IL 60618 USA; Department of Pharmacology, Toxicology and Pharmacy, Ludwig-Maximillians-University, Königinstr. 16, 80539 Munich, Germany; Department of Small Animal Medicine and Surgery, University of Veterinary Medicine Hannover, Bünteweg 9, 30559 Hannover, Germany; Department of Clinical Science and Services, Royal Veterinary College, Hatfield, AL9 7TA Hertfordshire UK

**Keywords:** Canine, Feline, Seizure, Imaging, Hippocampus

## Abstract

Epilepsy is one of the most common chronic neurological diseases in veterinary practice. Magnetic resonance imaging (MRI) is regarded as an important diagnostic test to reach the diagnosis of idiopathic epilepsy. However, given that the diagnosis requires the exclusion of other differentials for seizures, the parameters for MRI examination should allow the detection of subtle lesions which may not be obvious with existing techniques. In addition, there are several differentials for idiopathic epilepsy in humans, for example some focal cortical dysplasias, which may only apparent with special sequences, imaging planes and/or particular techniques used in performing the MRI scan. As a result, there is a need to standardize MRI examination in veterinary patients with techniques that reliably diagnose subtle lesions, identify post-seizure changes, and which will allow for future identification of underlying causes of seizures not yet apparent in the veterinary literature.

There is a need for a standardized veterinary epilepsy-specific MRI protocol which will facilitate more detailed examination of areas susceptible to generating and perpetuating seizures, is cost efficient, simple to perform and can be adapted for both low and high field scanners. Standardisation of imaging will improve clinical communication and uniformity of case definition between research studies. A 6–7 sequence epilepsy-specific MRI protocol for veterinary patients is proposed and further advanced MR and functional imaging is reviewed.

**Electronic supplementary material:**

The online version of this article (doi:10.1186/s12917-015-0466-x) contains supplementary material, which is available to authorized users.

## Background

Canine epilepsy has an estimated prevalence of 0.62–0.75 % in primary veterinary practice [1, 2] and as such is one of the most common chronic neurological diseases. Magnetic resonance imaging (MRI) is regarded as an essential diagnostic test however the specificity is limited because the diagnosis of idiopathic epilepsy is one of exclusion and the reliability of diagnosis is limited by available technology and expertise in interpretation. The International League against Epilepsy (ILAE) defines idiopathic epilepsy as *an epilepsy of predominately genetic or presumed genetic origin and in which there is no gross neuroanatomic or neuropathologic abnormality* [3]. Therefore by default, MRI examination of an animal with idiopathic epilepsy should be “normal” (in human epilepsy termed MRI–negative). However the ability to detect lesions depends on many factors that affect the quality of the MRI examination (Table 1). Some of these factors can be controlled, such as optimal slice thickness and sequence. Other factors are less easy to influence. For example, the ideal epilepsy protocol in humans (Table 2) would include a gradient echo or similar technique for detecting haemorrhage or calcification. However this sequence is sensitive to susceptibility artefacts arising from the skull bones for example the mastoid area of the temporal bone, which are a more significant problem in veterinary patients that have a greater bone:brain ratio than humans. The interface between bone and air can cause inhomogeneity in the magnetic field and signal void (susceptibility) artefact, particularly noticeable on special sequences such as diffusion-weighted imaging (DWI) and which can interfere with MR spectroscopic techniques.

**Table 1:** Factors that have an effect on the ability to detect epileptic lesions on MRI

Type	Example	Notes
Protocol	Slice thickness	Thinner slices give more chance of lesion detection. A routine scan with 5 mm thick slices and 0.5 mm interslice gaps with T1W and T2W transverse image acquisitions and gadolinium contrast enhancement may be adequate to evaluate gross cerebral abnormalities such as large tumours or malformations but may not detect subtle epileptic lesions. Slice thickness of 3 mm or less in at least 2 orientations is recommended for examination of the epileptic brain and larger slice size risks missing lesions less than 5 mm [38]. However MRI machines of 1 T or less cannot provide thin slices with sufficient SNR within reasonable time. For this reason machines under 1.5 T are considered insufficient for the imaging of human epilepsy patients unless there is no alternative [38].
	Sequence	Failure or inability to select the appropriate sequences to detect lesions. For example in humans, high resolution, volumetric and 3D MRI acquisition is recommended to obtained detailed information on hippocampal anatomy, cortical gyral patterns, improve grey and white matter contrast and to enable co-registration with other modalities or sequential MRI examinations [13, 38]. This requires a good quality machine (1.5 T or more) and careful orientation of slice plane relative to patient position. FLAIR sequence is regarded as the most useful image for detecting epileptic lesions in humans [38] however many low field machines produce FLAIR with low resolution.
Magnetic field strength	Low field versus high field	Imaging with higher magnetic field-strength provides improved signal-to-noise ratio and spatial resolution which allows shorter imaging times for a given resolution and/or higher resolution for a given imaging time. Higher signal-to-noise ratio allows better resolution with smaller voxel size and thinner slice thickness [7].
	1.5 T versus 3 T	
Coil	Type of coil used (for example Knee vs Head coil)	Coils with minimum distance between receiving coil and brain surface and minimal diameter increase SNR and therefore image quality. Some coils (for example brain coils) may limit the field of view that can be imaged before significant signal drop-off occurs. The lack of availability of dog-specific coils and variation in dog head size makes coil selection challenging in some cases.
	Available channels	An 8 channel brain coil is usual in veterinary MRI but a 32 channel brain coil will provide much better SNR and contrast resolution.
Operator factors	Inexperience / lack of training	A fully trained radiography technician understands the physics of MRI and anatomy allowing them to create images with excellent contrast and clarity and target the brain structures to be studied. Typically, a trained MRI technician has undertaken a 3-year radiography degree plus an additional 2–3 years of post-graduate MRI training. A poorly trained or unqualified operator may not be able to achieve optimal results from the machine that they have. In veterinary medicine it is possible to operate a MRI service without a specialist qualification.
	Diligence	There are ways of improving image quality, for example increasing the number of averages (NEX) however these tend to increase the acquisition time. Out with other reasons for decreasing imaging time (economic / duration of anaesthesia), operator motivation is a factor. Bearing this in mind any recommended epilepsy-specific MRI protocol should not be overly onerous in order to improve compliance. A basic protocol of 6 sequences is recommended [38].
Interpreter factors	Inexperience / lack of training	Failure to recognise significant lesions or over-interpretation of other features. A study in humans found that 61 % of epileptogenic lesions remained undetected following “non-expert” reports of “standard” MRI scans. The failure rate dropped to 9 % using an epilepsy tailored MRI protocol with interpretation by experienced neuro-radiologists [39].
Patient factors	Skull and air interface	In some machines may cause susceptibility artefacts on gradient echo and T1W 3D imaging
	Small brain	Slice thickness should be proportional to the brain volume to achieve images with diagnostic quality i.e. animals with smaller brain volume require thinner slices.
	Brain conformation	Changes in skull shape, in particular brachycephaly have resulted in changes in brain conformation [40].
	General anaesthetic	Increased time under general anaesthesia may increase risk to patient.
Economic factors	Time	Increased time of scanning increases cost and risks of anaesthesia. It is important to consider the balance between time of acquisition and image quality in an animal under general anaesthesia.
	Machine costs (purchase of hardware, software, housing and maintenance)	Imaging with higher magnetic field-strength allows for superior images in a shorter imaging time but at a greater cost.
	Relevance	Identification and localisation of epileptic lesion is vital in humans with drug-resistant epilepsy, who may be candidates for potentially curative resective epilepsy surgery. Whether this is applicable for dogs with idiopathic epilepsy remains to be seen. Technology that is only capable of detecting large structural pathology such as tumours may be sufficient if it does not alter the management. However acquisition of high quality scans may enable future identification of resectable lesions that are currently hypothesised.

**Table 2:** Epilepsy-specific MRI protocol for humans This “essential” 6 sequence protocol allows the detection of virtually all common epileptogenic lesion in humans and was proposed after systemic analysis of 2740 patients in a epilepsy pre-surgery program [13, 38, 41]

Human epilepsy-specific MRI protocol	
Slice thickness 3 mm or less	
➢ T2W - 2 sequence orientations for hippocampal angulation	
● Perpendicular to the long axis of the hippocampus	
● Along the long axis of the hippocampus	
➢ FLAIR - 2 sequence orientations for hippocampal angulation	
● Perpendicular to the long axis of the hippocampus	
● Along the long axis of the hippocampus	
➢ T1W	
● 3D volume with 1 mm isotropic voxel size	
➢ Hemosiderin/calcification sensitive sequences e.g. gradient echo	

The ability to detect epileptogenic lesions is further limited by economics. For example, imaging with a 3 tesla (3 T) MRI system gives better anatomical detail and is superior for detecting subtle lesions such as mesial temporal sclerosis [4] and migration disorders [5, 6]. However the initial and on-going cost of this technology is prohibitive for many institutions and indeed much of veterinary MRI is performed on low field (1 T or less) scanners, which have decreased spatial resolution and signal-to-noise ratio (SNR) [7].

Other technology may need to be employed to detect lesions in MRI–negative patients. Methods of processing MRI data post-acquisition have identified previously undetectable or overlooked abnormalities in humans [8, 9]. One such example is employed to improve hippocampal volumetric measurements in the sparsely myelinated and small brain of neonatal humans. To achieve this, contrast is optimised by combining dual echo T2W and proton density images [10]. In large part this is based upon the fact that discovery of a surgically resectable lesion significantly improves the prognosis in human drug-resistant focal epilepsy, including abnormalities of the hippocampus in the region of the mesial temporal lobe. As a result, if the MRI is negative then further work-up, for example with functional MR imaging, is engaged to help localize the epileptogenic lesion [11–13]. Table 3 details examples of the modalities used, none of which are established as routine in animals. However before making recommendations for advanced imaging, the veterinary surgeon and the owner must be clear about what is to be gained. Unless the diagnostic procedure changes the outcome or management there may be little achieved by subjecting an animal to invasive and/or expensive procedures. For example, Smith and others found that if an epileptic dog was less than six years old and had a normal inter-ictal neurological examination then there was a 97 % confidence of a unremarkable low field brain MRI, making diagnosis of idiopathic epilepsy very likely [14]. At present, given the lack of surgical or other therapeutic techniques available to improve prognosis over standard antiepileptic therapy, more research is required to improve the diagnostic sensitivity of MRI and establish the value of such therapeutic techniques.

**Table 3:** Novel imaging modalities for identifying epileptic foci

Modality	Principle	Veterinary application
Magnetoencephalography (MEG) and magnetic source imaging (MSI)	MEG – non-invasive functional imaging recording magnetic flux on the head surface associated with electrical currents in activated sets of neurons. MSI - created when MEG data is superimposed on a MRI [42].	Has been performed experimentally in anaesthetised non-epileptic dog [43].
		May be limited by requirement for anaesthesia [44].
		Identity microchip may interfere with recording [45].
		Requires a magnetically shielded room and other expensive equipment [12].
Positron Emission Tomography (PET)	Functional representational of brain activity (dependent of the radionuclide tracer utilised) e.g. local glucose utilisation (fluorine-18 fluorodeoxyglucose - FDG). Brain regions containing the epileptogenic zone have hypometabolism on inter-ictal FDG-PET [12]. PET and MRI co-registration or integrated PET/MR with simultaneous acquisition is considered superior [8].	FDG-PET may be useful as a diagnostic test for idiopathic epilepsy in the dog [46, 47]
Ictal and inter-ictal single-photon emission computed tomography (ictal/inter-ictal SPECT)	Injection of a radiolabeled tracer during ictus and inter-ictus. Statistical comparison of the blood flow changes. Ideally co-registered to MRI (SISCOM) [48, 49].	Practical difficulties of performing in ictus. Has been performed in inter-ictus and in one study demonstrated subcortical hypoperfusion in epileptic dogs [50]
Diffusion tensor imaging (DTI)	Detects tissue microstructural pathology that influences freedom of water molecular diffusion. Has been used to detect hippocampal and temporal lobe pathology in TLE and DTI tractography has been used in surgical planning [12]. Has demonstrated microstructural alterations in large white matter tracts in idiopathic generalised epilepsy [51]	Experimental studies suggest DTI is feasible in dog [52–54] and structural abnormalities have been identified in a compulsive behaviour disorder [55]. No application for epilepsy yet.
Functional magnetic resonance imaging (fMRI)	Utilises the different magnetic susceptibilities of deoxygenated and oxygenated haemoglobin (blood oxygenation level dependent (BOLD) contrast). Deoxygenated haemoglobin is paramagnetic leading to distortion of magnetic fields and a shorter T2 relaxation time. Areas of increased brain activity have greater metabolic demand and more oxygenated haemoglobin and a prolonged T2 relaxation time. The difference in BOLD at rest and during a specific task (such as language and memory) indicates the areas of the brain activated by the task [12].	Laboratory experimental studies, none relating to epilepsy [56]. Has been used in trained awake dogs to assess cognition [57–59].
fMRI-EEG	EEG is acquired using a specialized system in the MRI machine while acquiring a blood oxygenation level dependent (BOLD) sequence. The EEG is analysed for epileptiform discharges spikes and the corresponding BOLD fMRI change is evaluated [12].	None as yet
Functional connectivity MRI (FcMRI)	Utilizes the principles of fMRI to demarcate brain networks. It evaluates the structural changes distant from the epileptic focus. Main application is in pathophysiology of the epilepsy but has the potential to guide surgery [12].	None as yet
Near infra-red spectroscopy (NIRS)	Probe transmits near infra-red spectrum wavelength rays that passed through the cranium to a depth of approximately 2 cm and is absorbed by haemoglobin in the tissue. Reflected rays are detected by a sensor probe. The strength of reflected rays is inversely related to the concentration of haemoglobin in the brain tissue. The resulting images are co-registered to the MRI to lateralize and localize the signal changes [12].	Pilot studies performed assessing positive emotional states in dogs [60]
		Limited to superficial brain structures. May have limited application in dogs with thicker skulls and muscle. However can be performed in awake animals.
Magnetic resonance spectroscopy (MRS)	MRS can be used to measure creatine (Cr), N-acetyl aspartate (NAA), choline (Cho), lactate, myo-inositol and GABA non-invasively in the brain tissue [12]. Reduced NAA/Cho and NAA/Cr was found in the lesional temporal lobe in TLE [61] and in the epileptogenic/irritative zone in frontal lobe epilepsy [62]. These MRS changes were most likely due to cell dysfunction than cell loss [12]	Studies in healthy dogs [63], laboratory canine model of seizures [64] and in some disease states [65].
Arterial spin labelling (ASL)	ASL is a non-invasive MRI technique to assess brain perfusion and therefore image functional areas of the brain. Arterial blood is magnetically labelled using a 180° radio frequency (RF) inversion pulse prior to imaging the region of interest (ROI). The labelled blood flows into the ROI and reduces the MR signal and image intensity at this area. Subtracting this image from the baseline MRI creates the perfusion image which reflects the amount of blood delivered to each voxel [12, 66]. It has been used to show mesial temporal hypometabolism [67] and hippocampal volume loss [68]	None as yet

The purpose of this article is to propose an epilepsy-specific MRI protocol that will optimise detection of lesions ruling out idiopathic epilepsy as a diagnosis, standardise the diagnosis for entry into clinical trials and facilitate detection of lesions which develop as a consequence of epilepsy, as well as provide high quality data for future studies investigating the pathophysiology of epilepsy.

### Aim of advanced diagnostic imaging for animals with epilepsy

There are three main aims of advanced diagnostic imaging of the epileptic animal: 1) to rule out causes of epileptic seizures which may be treatable with means other than antiepileptic therapy only (e.g. inflammatory or infectious brain disease) 2) to identify lesions which are caused by seizures but are not themselves the source of seizures for example hippocampal sclerosis and 3) to provide data to further advance the field of research into the pathogenesis and/or treatment of epilepsy. Importantly, MRI must always be preceded by a thorough investigation including a good clinical history with clinical and neurological examination (see Consensus Proposal on the diagnostic approach to epilepsy in dogs). In addition, the absence of lesions identifiable on MRI examination does not indicate prognosis or which drugs are most appropriate. However MRI may enable the detection of lesions that may be associated with drug-resistance such as hippocampal sclerosis [5]. High resolution imaging of the hippocampus is therefore paramount in humans but the value of this remains undetermined in animals [15, 16].

### Identification of the epileptogenic lesion

Most veterinary hospitals that offer advanced diagnostic imaging use the same protocol for the epileptic brain as for detection of gross intracranial pathology such as tumours. This reflects the aim of the procedure, namely to identify those lesions that have a different prognosis or treatment to idiopathic epilepsy. In human medicine, different MRI protocols are performed depending on whether the patient is expected to have idiopathic or structural epilepsy. Some might recommend that epileptic animals that are not expected to have idiopathic epilepsy (for example those animals younger than 6 months or older than 6 years or those patients with abnormal inter-ictal neurological examination) could be examined using an MRI protocol that does not require as high a resolution imaging of the brain while those patients expected to have idiopathic epilepsy could be examined using a higher resolution protocol. However in practice the expense and risk associated with general anaesthesia in veterinary patients makes it unlikely that more than one protocol be used for scanning an animal with epileptic seizures. Therefore any protocol developed for animals must be capable of diagnosing both types of epilepsy.

For animals with a probable diagnosis of idiopathic epilepsy (i.e. those animals that fulfil Tier 1 level of confidence for diagnosis - see Consensus Proposal: Diagnostic approach to epilepsy in dogs), many of the differential diagnoses associated with structural epilepsy, in particular large malformations and neoplastic causes, are relatively straightforward to identify [6, 17, 18]. However, several are associated with subtle changes that may be easily missed without adequate resolution scanning and careful interpretation. The most common of these are listed in Table 4. It must also be remembered that any lesion identified is not automatically epileptogenic in nature and other evidence (e.g. EEG, seizure history) may be required to demonstrate this [19].

**Table 4:** Differentials for idiopathic epilepsy that may require high resolution imaging to identify

Condition	Imaging features	References
Congenital and developmental causes		
Nodular heteroptopia/ focal cortical dysplasia	Abnormal location or thickness of deep grey matter, commonly periventricular or interspersed amongst white matter.	[69]
L2-hydroxyglutaric aciduria	Poor distinction between grey and white matter throughout cerebral hemispheres and deep grey matter. Bilateral grey matter hyperintensity, especially the thalamus and cerebellum	[70]
Infectious and inflammatory causes		
Distemper encephalitis	Patchy, asymmetric T2-weighted hyperintensities with mild or no contrast enhancement on T1W scans. Lesions are usually asymmetric, large, round to ovoid in shape throughout different parts of the forebrain, especially in grey matter of the temporal lobe, as well as the brainstem, cerebellum and subcortical white matter.	[71]
Rabies encephalitis	Very mild lesions - bilaterally symmetrical T2W hyperintensities in temporal lobes, hippocampus, hypothalamus, midbrain and pons with little or no contrast enhancement.	[72]
Metabolic, endocrine and nutritional causes		
Hepatic encephalopathy	Bilaterally symmetrical T1W hyperintensities in caudate nuclei, thalamus, not associated with contrast enhancement	[73]
Thiamine deficiency	Bilateral, symmetric T2W hyperintensities in caudate nuclei, lateral geniculate nuclei, red nucleus, caudal colliculi, facial and vestibular nuclei	[74]

### Identification of lesions which are the consequence of seizures

**Fig. 1:**
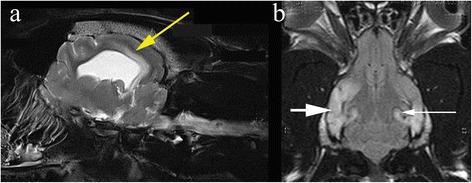
Post-ictal changes in the temporal and parietal lobe. Images obtained in a 1.5 T Siemens Symphony, Erlangen, Germany. Post-ictal oedema in the temporal lobe (*short white arrow*), hippocampus (*long white arrow*) and cingulate gyrus (*yellow arrow*) in a 2 year male English Bulldog that presented in status epilepticus

**Fig. 2:**
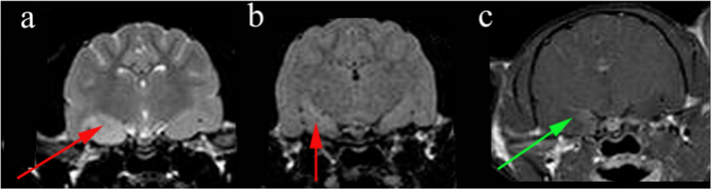
Hippocampal changes in an 8 month male neutered Oriental Shorthair presented with status epilepticus. **a** Transverse TW2 at level of pituitary gland. There is hyperintensity of the right temporal lobe (red arrow) (**b**) Transverse FLAIR at level of pituitary gland also demonstrating hyperintensity of the right temporal lobe (red arrow) (**c**) Transverse TW1 at level of pituitary gland. There is slight gadolinium contrast enhancement in the mesial temporal lobe. Images reproduced with the kind permission of Dr Ane Uriarte . The cat was suspected to have limbic encephalitis

Longitudinal studies of epileptic humans suggest that 10 % of newly diagnosed patients and 25 % of those with chronic active epilepsy develop significant cerebral, hippocampal or cerebellar atrophy over 3.5 years [20]. More acute changes secondary to seizures have also been reported (Fig. 1) and it is important that imaging techniques are able to differentiate these resultant, reversible changes from those that may be the cause of seizures. Most commonly, changes that are the result of seizures are found as T2-weighted hyperintensities predominantly in the piriform and temporal lobes, as well as the cingulate gyrus and hippocampus [21]. These changes resemble those reported in humans and are likely to represent a mixture of cytotoxic oedema and gliosis [21]. In some cases mild contrast uptake may also be apparent [22]. In general these changes are diffuse, relatively extensive, and their characteristic location makes it straightforward to distinguish them from epileptogenic lesions with either high-field or low-field scanners. However sometimes in can be difficult to ascertain if the changes are cause or effect for example in VGKC-complex/LGI1 antibody-associated limbic encephalitis in cats (Fig. 2) [23]. Cerebrospinal fluid analysis can be unhelpful because post-ictal pleocytosis can occur [24]. In ideal circumstances it would be preferred to repeat imaging in the post ictal period and also assess changes in brain volume/ atrophy however available finances can limit this opportunity. In those patients with whom some doubt may remain, however, the most useful procedure for identifying post-ictal MRI changes is to repeat the scan at a later date, since these changes resolve usually within 16 weeks [21].

### Providing data for further research into the pathogenesis and treatment of seizures

In humans, much attention has focussed on the hippocampus because temporal lobe epilepsy (TLE) is the most common cause of complex focal epilepsy, and mesial temporal sclerosis (i.e. severe neuronal cell loss and gliosis in the medial portion of the temporal lobe and particularly in the hippocampus) is a major pathological finding, occurring in roughly 50 % of TLE patients [25]. The pathogenesis of mesial temporal sclerosis is multifactorial and includes genetic factors and molecular events such as channelopathies, activation of NMDA receptors, and other conditions related to Ca(2+) influx into neurons and imbalance of Ca(2+)-binding proteins [26]. There has been much debate as to whether these changes are the cause or the effect of seizures. Most significantly, the surgical removal of these regions in patients with an electroencephalographic (EEG) diagnosis that confirms their location as the source of seizure activity results in significant improvement in seizure control in up to 80 % of patients [27, 28]. The current diagnosis of hippocampal sclerosis in humans requires specific positioning of slices in order to define the hippocampus accurately, together with a considerable body of research defining the range of normal volumes in healthy individuals. These techniques for hippocampal measurement have been established for many years and TLE is one of the more common homogenous forms of epilepsy, so adequate numbers of patients are available for studies [20].

**Fig. 3:**
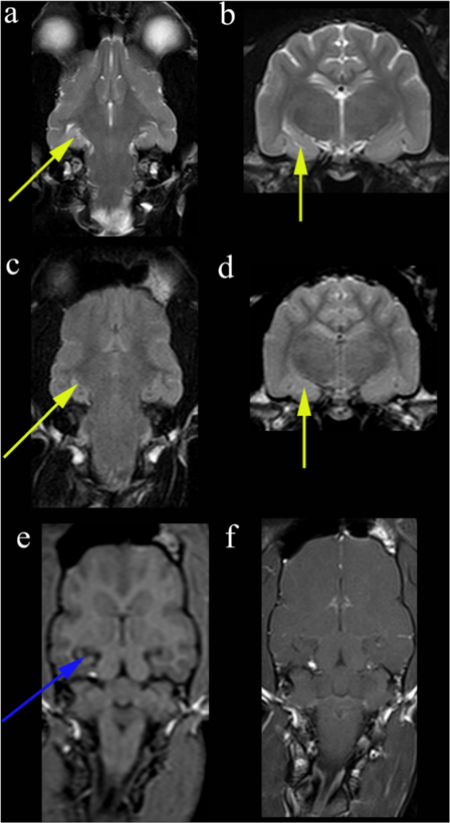
Hippocampal changes in a 22 month male neutered Oriental Shorthair with drug-resitant epilepsy. Images obtained in a 1.5 T MRI (Siemens Symphony, Erlangen, Germany) 12 months after the images in Fig. 2. Despite an initial course of corticosteroids and polypharmacy with multiple anti-convulsants the cat seizured on an almost daily basis. **a** Dorsal T2W orientated perpendicular to long axis of the hippocampus. **b** Transverse T2W orientated parallel to the long axis of the hippocampus. **c** Dorsal FLAIR orientated perpendicular to long axis of the hippocampus. **d** Transverse FLAIR orientated to long axis of the hippocampus. **e** Dorsal T1W 3D images 1 mm slice thickness orientated perpendicular to long axis of the hippocampus. **f** Dorsal T1W orientated perpendicular to long axis of the hippocampus post gadolinium. On FLAIR and T2W images there is reduction in volume and a hyperintensity of the hippocampus (*yellow arrows*). With the TIW 3D images it is possible to appreciate loss in definition between the white and grey matter in addition to reduction in volume of the hippocampus (*blue arrow*) There is no abnormal enhancement with gadolinium contrast

Whether hippocampal volume loss and mesial temporal sclerosis is a parameter that should be assessed in dog has yet to be established (Fig. 1). Hippocampal atrophy has been demonstrated in rodent models [29] and in familial spontaneous epileptic cats where EEG features suggested TLE [16]. Reduced volume of the hippocampus / hippocampal atrophy has been demonstrated in epileptic dogs [15]. Furthermore histopathological changes consistent with hippocampal sclerosis have been well described in epileptic cats [28, 30, 31] (Figs. 2, 3). For these reasons, as well as the recognition that hippocampal sclerosis represents a common surgical target in the treatment of human epilepsy, it appears prudent to evaluate the hippocampus accurately in animal patients with epilepsy. Therefore routine MR evaluation of the epileptic subject should at least include a visual assessment of the hippocampus for atrophy, asymmetry in size, loss of defined morphologic structure, increased T2W or T2W Fluid Attenuated Inversion Recovery (FLAIR) signal and decreased T1W signal [15, 32]. Hippocampal T2W hyperintensity is well correlated with pathology and hippocampal sclerosis and measurement of the T2 relaxation time (T2 relaxometry) can provide an objective measure in humans but has not been assessed in dogs or cats [32]. There is an argument that volumetric studies should be performed in veterinary patients (Table 5) and recent studies have defined the range in normal animals [33]. However making volumetric measurements is a labour intensive process requiring high resolution MRI and personnel training [33]. Currently this is only used as a research tool, although in the future automated atlas-based segmentation may make hippocampal volumetry more routine. Even in humans where hippocampal volumetry has established utility, the time demands and required technical skills mean that it has been difficult to integrate into clinical practice [34]. Consequently patients with a surgically resectable lesion may be missed. This has led to the development of automated software which will compare an individual patient’s regional brain volumes with a normative database, correcting for sex, head size, and age [34]. Establishing automated software in veterinary patients is challenging due to difficulties in automatic brain extraction algorithms arising from the great variation is head shape and brain size and conformation. Establishment of reference ranges for the three basic canine brain shapes (dolicocephalic, mesaticephalic and brachycephalic) may represent a suitable compromise. Before making a recommendation of measurement of hippocampal volumes in veterinary patients it should be remembered that hippocampal sclerosis is not applicable to all idiopathic generalised epilepsies in humans especially if the epileptogenic focus is not the temporal lobe [35]. Repeated seizures will affect other structures pathologically including the amygdala, cerebral neocortex and the cerebellum [20].

**Table 5:** Reasons why it may be appropriate to perform volumetric studies on hippocampus or other potentially epileptogenic areas

Rationale for volumetric analysis	
** ➢ To establish normative date**	
● Breed and size variations	
● Age	
● Gender	
● Within subject functional and anatomical asymmetry [75]	
** ➢ To provide a baseline**	
● At initial diagnosis and for serial comparison, for example, if develops drug-resistant epilepsy	
** ➢ To identify patients with poor prognosis / less likely to respond to treatment**	
● Volume compared to normative data	
● Within subject asymmetry in volume	
**➢ Improving cohort selection for entry into clinical trials evaluating**	
● Antiepileptic drugs	
● Neuro-protective agents that may modulate the consequences of epilepsy on cognition and behaviour [76]	
● Novel treatment modalities	

### Existing MRI protocols

Current protocols vary substantially between institutions. Polling of members of the international veterinary epilepsy task force determined that all protocols currently include imaging in at least two orientations (transverse and sagittal) and the majority in three planes (dorsal, typically orientated parallel to hard palate rather than perpendicular to the long axis of the hippocampus). T2W, T2W FLAIR and T1W images pre and post paramagnetic contrast (gadolinium based) are included as standard in most protocols used by specialists who are active in the veterinary field. This differs from human epilepsy-specific MRI protocols where routine administration of gadolinium contrast is considered to provide little advantage for idiopathic or TLE and is reserved for patients in whom there is concern for tumour, vascular malformations, inflammation, and infectious disease or when these are suspected based on review of non-contrast studies [35]. Routine administration of gadolinium contrast in veterinary medicine has been questioned [36]. Other sequences currently included in “veterinary brain protocols” vary between institutions and may include Gradient Echo (GE), T1 weighted Inversion Recovery (T1WIR), Diffusion Weighted Imaging (DWI) and Short Tau Inversion Recovery (STIR) or other fat suppression techniques.

This variation between institutions suggests a need for a uniform veterinary epilepsy-specific MRI protocol that can provide a solid platform for clinical communication and comparability of case definition between research studies. There is also an argument for an MRI protocol that is optimized for epilepsy evaluation facilitating more detailed examination of areas susceptible to generating and perpetuating seizures such as the frontal and temporal lobes and other structures likely to be evaluated at post-mortem in patients who have died. Such a protocol must acknowledge financial constraints, be tailored for low or high field machines and also complement pathological studies.

### Consensus on epilepsy-specific MRI protocol

**Fig. 4:**
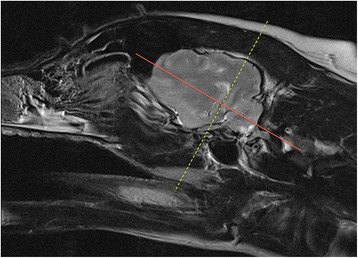
Parasaggital slice in a veterinary epilepsy-specific protocol for 1.5 T MRI scanner. T2W parasagittal image of the brain demonstrating a planned sequence parallel (*yellow dotted line*) and perpendicular (*red solid line*) to the long axis of the hippocampus. Images obtained in a 1.5 T MRI (Siemens Symphony, Erlangen, Germany)

**Fig. 5:**
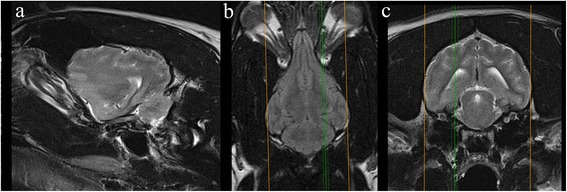
Veterinary epilepsy-specific protocol for high field MRI. Images obtained in a 1.5 T MRI (Siemens Symphony, Erlangen, Germany). Triplet of MR images illustrating the positon of the parasagittal slice containing the hippocampus. *Left*. T2W parasagittal section demonstrating the hippocampus for sequences orientated relative to the long axis. *Middle*. Dorsal FLAIR of the brain at the level of the orbits illustrating the position of the parasagittal slice (*green line*). *Right* T2W transverse of the brain at the level of the hippocampus illustrating the position of the parasagittal slice (*green line*)

**Fig. 6:**
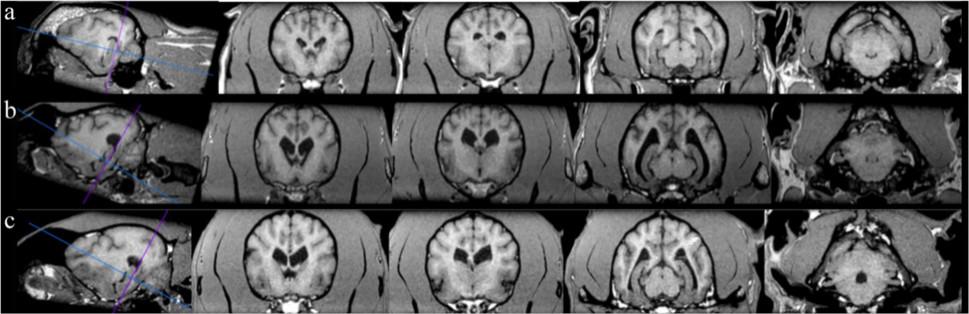
Variation in appearance of the hippocampus in different skull shapes. **a** brachycephalic vs (**b**) mesocephalic vs (**c**) dolicocephalic with orientation of transverse scans parallel to the long axis of the hippocampus

**Fig. 7:**
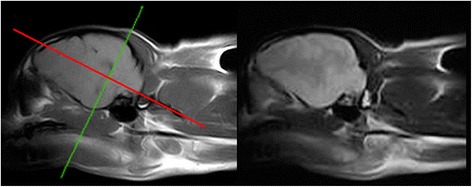
Veterinary epilepsy-specific protocol for low field MRI. T1W parasagittal image (*left*) of the brain demonstrating a planned sequence orientated parallel (*green line*) and perpendicular (*red solid line*) to the long axis of the hippocampus. It is easier to identify the hippocampus in T1W images from a low field machine. For comparison the corresponding T2W parasagittal images are included (*right*). Images obtained in 0.2 T MRI (Esaote Grande, Genova, Italy)

**Fig. 8:**
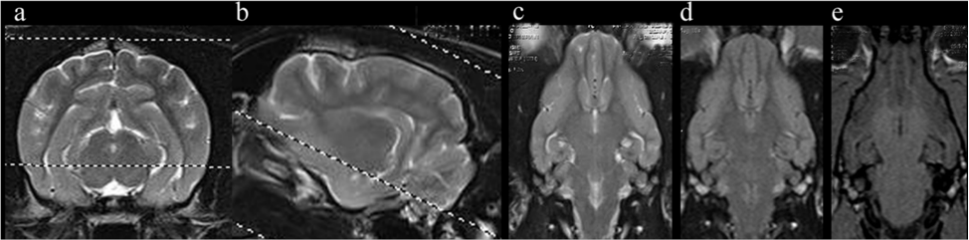
Veterinary epilepsy-specific protocol for high field MRI. The imaging time for 6 sequences (Figs. 8 and 9) on a 1.5 T MRI was 45 min. The subject was an epileptic 16 month female Cocker spaniel (**a**) and (**b**) Transverse and parasagittal T2W image illustrating slice orientation. **c** Dorsal T2W orientated perpendicular to long axis of the hippocampus (**d**) Dorsal FLAIR orientated perpendicular to long axis of the hippocampus (**e**) Dorsal T1W 3D images1mm slice thickness orientated perpendicular to long axis of the hippocampus

**Fig. 9:**
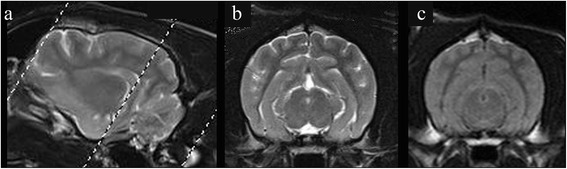
Veterinary epilepsy-specific protocol for high field MRI. **a** parasagittal T2W image illustrating slice orientation. **b** Transverse T2W orientated parallel to the long axis of the hippocampus. **c** Transverse FLAIR orientated parallel to the long axis of the hippocampus. Images obtained in a 1.5 T MRI (Siemens Symphony, Erlangen, Germany)

**Fig. 10:**
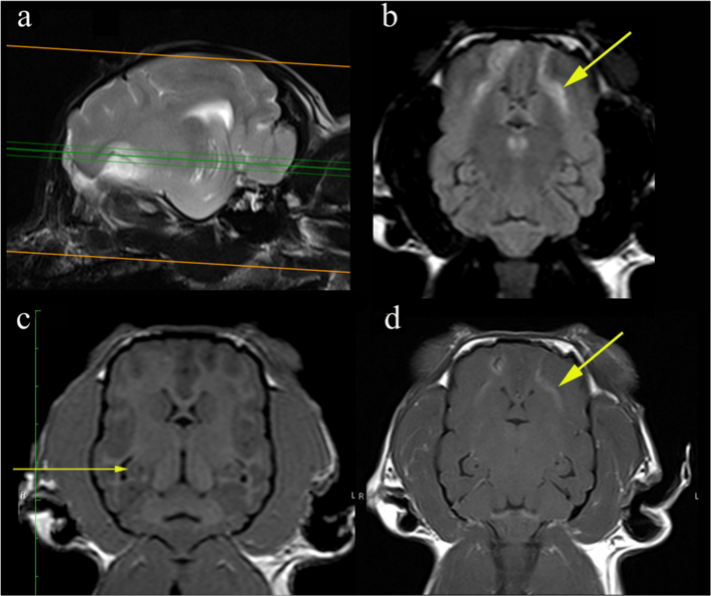
Representative MRI from a 2.95 kg 5 year female entire Chihuahua dog that underwent a diagnostic investigation for cluster seizures. **a** Parasagittal image demonstrating the hippocampus and the planned imaging perpendicular to the long axis (**b**) Dorsal FLAIR images orientated perpendicular to long axis of the hippocampus demonstrating hyperintensity in the frontal lobe (*short arrow*). Although this protocol is optimised for detection of hippocampal lesions visualisation of other pathology is not compromised. **c** Dorsal T1W 3D images 1 mm slice thickness orientated perpendicular to long axis of the hippocampus. The scrolled structure of the hippocampus is clearly defined despite the small patient size. Furthermore the demarcation between white and grey matter can be appreciated (*long arrow*). **d** Post gadolinium T1W images are obtained in further investigation of the frontal lobe pathology. The patient was diagnosed subsequently with necrotising encephalitis. Images obtained in a 1.5 T MRI (Siemens Symphony, Erlangen, Germany)

There is a need for a standardized veterinary epilepsy-specific MRI protocol which will facilitate more detailed examination of areas susceptible to generating and perpetuating seizures, complement pathological studies, is economical, simple to perform and can be adapted for both low and high field machines. Standardisation of imaging will improve clinical communication and uniformity of case definition between research studies. We propose the following protocols (Tables 6 and 7). During protocol set-up, it is recommended that different parameters (such as flip angle) are trialed in order to obtain the optimal balance between grey-white matter contrast and SNR (for information on MR parameters for 0, 2, 1.5 and 3T see Additional files 1, 2 and 3). Both protocols start with obtaining a sagittal sequence. Due to the difference in anatomical definition this is a T2W sequence in high field machines and T1W sequence in low field machines. In addition to identifying gross structural pathology the sagittal images allow assessment of cerebellar atrophy according to the protocol described by Thames and others [37]. Using parasagittal images the long axis of the hippocampus is identified (Figs. 4, 5, 6, 7, 8 and 9). The hippocampus forms the medial wall of the temporal horn of the lateral ventricle and is delineated on parasagittal images by the contrasting cerebrospinal fluid. After identification of the hippocampus, T2W and sequences are orientated parallel and perpendicular to the long axis of the hippocampus (Figs. 4 and 7). T2W and FLAIR are acknowledged to be optimal for detection of epileptic lesions in humans in particular hippocampal changes (Figs. 2 and 3) and therefore in humans two FLAIR sequences would be obtained [38], however, it is recognized that performing two FLAIR sequences may increase scanning time significantly therefore we recommend that at a minimum a dorsal FLAIR sequence perpendicular to the long axis of the hippocampus is obtained with an option for an additional transverse sequence parallel to the long axis of the hippocampus. In high field scanners a transverse gradient echo or similar sequences sensitive to detection of hemosiderin and / or calcification should be obtained. Like the other images this transverse image is also orientated parallel to the hippocampus. In low field scanners additional T1W sequences are recommended (Table 5). Some high field machines may be able to obtain good resolution 3D TW1 images (Figs. 3, 8 and 10). For these the acquired slice thickness is 1 mm or less giving improved chance of lesion detection, better white and grey matter definition and can be processed after imaging into any anatomical plane including oblique. Furthermore this will facilitate volumetric measurements and to enable co-registration with other modalities or sequential MRI examinations [13, 38]. If this is not possible then a dorsal T1W sequence oriented along the long axis of the hippocampus is suggested. As indicated above there is an argument against routine paramagnetic contrast administration however it is acknowledged that many veterinary neurologists would feel a MRI study of an epileptic patient was incomplete without this therefore these sequences are an optional extra. However if pathology was detected in the unenhanced study, post-gadolinium sequences would be indicated (Fig. 10). Recommended slice thickness is 3 mm or less for high field machines and 4 mm or less for low field machines. Such a protocol would give 6–7 sequences for a high field machine and 6–7 sequences on a low field machine (not including optional paramagnetic contrast enhancement).

**Table 6:** Proposed epilepsy-specific MRI protocol for a high field machine

Veterinary epilepsy-specific protocol for 1.5 T MRI	
Slice thickness 3 mm or less	
**➢ T2W – 3 sequence orientations**	
● Sagittal enabling identification long axis of the hippocampus	
● Dorsal, perpendicular to the long axis of the hippocampus	
● Transverse, parallel to the long axis of the hippocampus	
**➢ FLAIR 1–2 sequence orientations for hippocampal angulation**	
● Dorsal, perpendicular to the long axis of the hippocampus	
● Transverse, parallel to the long axis of the hippocampus (optional)	
** ➢ T1W**	
● 3D technique at 1 mm isotropic voxel size (if possible) or routine T1W dorsal, perpendicular to long axis of the hippocampus	
● T1W post paramagnetic contrast injection enhancement if indicated by other pathology / desired by clinician	
**➢ Hemosiderin / calcification sensitive sequences e.g. gradient echo**	
● Transverse, parallel to the long axis of the hippocampus

**Table 7:** Proposed epilepsy specific MRI protocol for a low field machine

Veterinary epilepsy-specific protocol for 0.2 T MRI	
Slice thickness 4 mm or less	
**➢ T1W – 3 sequence orientations**	
● Sagittal enabling identification of the long axis of the hippocampus	
● Dorsal, perpendicular to the long axis of the hippocampus	
● Transverse, parallel to the long axis of the hippocampus	
** ➢ T2W - 2 sequence orientations for hippocampal angulation**	
● Dorsal, perpendicular to the long axis of the hippocampus	
● Transverse, parallel to the long axis of the hippocampus	
**➢ FLAIR 1–2 sequence orientations for hippocampal angulation**	
● Dorsal, perpendicular to the long axis of the hippocampus	
● Transverse parallel to the long axis of the hippocampus (optional)	
**➢ T1W post paramagnetic contrast enhancement**	
● If indicated by other pathology / desired by clinician	
● Number of sequences determined by pathology

## Additional files

Additional file 1:
**MRI Parameters for epilepsy-specific protocol on a 0.2 T machine.**
Additional file 2:
**MRI Parameters for epilepsy-specific protocol on a 1.5 T machine.**
Additional file 3:
**MRI Parameters for epilepsy-specific protocol on a 3 T machine.**

## Abbreviations

MRI: Magnetic resonance imaging
MR: Magnetic resonance
ILAE: International League Against Epilepsy
IVETF: International Veterinary Epilepsy Task Force
SNR: Signal-to-Noise-Ratio
TLE: Temporal lobe epilepsy
FLAIR: Fluid attenuated inversion recovery
GE: Gradient echo
T1WIR: T1 weighted inversion recovery
DWI: Diffusion weighted imaging
STIR: Short tau inversion recovery
