# Supplementary material for: International Veterinary Epilepsy Task Force recommendations for a veterinary epilepsy-specific MRI protocol
Source: BMC Vet Res. 2015 Aug 28;11:194. doi: 10.1186/s12917-015-0466-x (PMC4594743; doi:10.1186/s12917-015-0466-x)
Supplement: Additional file 1: — MRI Parameters for epilepsy-specific protocol on a 0.2 T machine. [file 12917_2015_466_MOESM1_ESM.docx]

**Additional file 1: MRI Parameters for Epilepsy protocol on a 0.2T machine** (0.2 Tesla Airis Mate, Hitachi Medical Corp, Tokyo, Japan)

|  |  | TR | | TE | | FOV | | Matrix resolution | | Flip angle | | Slice thickness / gap | | NEX | | IR time | | Time of Acquisition | |
| --- | --- | --- | --- | --- | --- | --- | --- | --- | --- | --- | --- | --- | --- | --- | --- | --- | --- | --- | --- |
|  |  | Small | Large | Small | Large | Small | Large | Small | Large | Small | Large | Small | Large | Small | Large | Small | Large | Small | Large |
| 1 | T2W  Sag | 3400 | 3900 | 120 | 120 | 150 | 200 | 256 | 256 | 90 | 90 | 3/no gap | 4/no gap | 4 | 4 |  |  | 9:03 | 8:19 |
| 2 | T2W  Tra | 4600 | 7200 | 120 | 120 | 150 | 200 | 256 | 256 | 90 | 90 | 3/no gap | 4/no gap | 4 | 4 |  |  | 12:30 | 15:22 |
| 3 | T1W  Sag | 300 | 400 | 27 | 27 | 150 | 200 | 256 | 256 | 90 | 90 | 3/no gap | 4/no gap | 2 | 2 |  |  | 2:41 | 4:10 |
| 4 | T1W  Tra | 450 | 700 | 27 | 27 | 150 | 200 | 256 | 256 | 90 | 90 | 3/no gap | 4/no gap | 2 | 2 |  |  | 5:10 | 6:15 |
| 5 | T1W  Dor | 300 | 400 | 27 | 27 | 120 | 200 | 256 | 256 | 90 | 90 | 3/no gap | 4/no gap | 2 | 2 |  |  | 2:29 | 3:18 |
| 6 | FLAIR  Tra | 8000 | 11700 | 90 | 90 | 150 | 200 | 256 | 256 | 90 | 90 | 3/no gap | 4/no gap | 2 | 2 | 1870 | 1970 | 11:0 | 12:29 |
|  |  |  |  |  |  |  |  |  |  |  |  |  |  |  |  |  |  | 42:23 | 49:13 |
