# Supplementary material for: International Veterinary Epilepsy Task Force recommendations for a veterinary epilepsy-specific MRI protocol
Source: BMC Vet Res. 2015 Aug 28;11:194. doi: 10.1186/s12917-015-0466-x (PMC4594743; doi:10.1186/s12917-015-0466-x)
Supplement: Additional file 2: — MRI Parameters for epilepsy-specific protocol on a 1.5 T machine. [file 12917_2015_466_MOESM2_ESM.docx]

**Additional file 2 MRI Parameters for epilepsy – specific protocol on a 1.5T machine**

|  |  | TR | | TE | | FOV | | Matrix resolution | | Flip angle | | Slice thickness / gap | | NEX | | IR time | | Time of Acquisition | |
| --- | --- | --- | --- | --- | --- | --- | --- | --- | --- | --- | --- | --- | --- | --- | --- | --- | --- | --- | --- |
|  |  | Small | Large | Small | Large | Small | Large | Small | Large | Small | Large | Small | Large | Small | Large | Small | Large | Small | Large |
| 1 | T2W Sag | 2700 | 3200 | 115 | 110 | 150 | 200 | 384 | 320 | 150 | 150 | 3/0.3 | 3./0.3 | 3 | 3 |  |  | 6:14 | 5:57 |
| 2 | T2W Tra | 3700 | 4200 | 117 | 110 | 120 |  | 384 | 320 | 150 | 150 | 3.5/0.35 | 3 /0.3 | 2 | 2 |  |  | 4:00 | 4:45 |
| 3 | T2W Dorsal |  | 3700 |  | 90 |  | 200 |  | 320 |  | 150 |  | 3/ 0.3 |  | 2 |  |  |  | 3.31 |
| 4 | FLAIR Tra | 8000 | 8000 | 125 | 125 | 120 | 200 | 512 | 512 | 150 | 150 | 3.5 /0.35 | 3 /0.3 | 3 | 2 | 2500 | 2500 | 6:20 | 4:48 |
| 5 | FLAIR Dor | 8000 | 8000 | 123 | 125 | 120 | 200 | 512 | 512 | 150 | 150 | 3 /0.3 | 3 /0.3 | 3 | 2 | 2500 | 2500 | 4:54 | 4:40 |
| 6 | T1W 3D Dor | 1900 | 1900 | 3.93 | 3.93 | 120 | 180 | 256 | 256 | 15 | 15 | 1 | 1 | 2 | 2 | 1100 | 1100 | 7:40 | 7:40 |
| 7 | T1W Tra | 500 | 520 | 13 | 12 | 120 | 180 | 320 | 384 | 90 | 90 | 3.5 /0.35 | 3 /0.3 | 3 | 3 |  |  | 4:26 | 5:22 |
|  |  |  |  |  |  |  |  |  |  |  |  |  |  |  |  |  |  | 32:54 | 35:23 |
