# Supplementary material for: International Veterinary Epilepsy Task Force recommendations for a veterinary epilepsy-specific MRI protocol
Source: BMC Vet Res. 2015 Aug 28;11:194. doi: 10.1186/s12917-015-0466-x (PMC4594743; doi:10.1186/s12917-015-0466-x)
Supplement: Additional file 3: — MRI Parameters for epilepsy-specific protocol on a 3 T machine. [file 12917_2015_466_MOESM3_ESM.doc]

Additional file 3: Sequences developed on 3T Siemens machine using a 32 channel brain coil.

Note: in addition to these we acquire MRS and DWI for white matter tractography; for this reason we do not do additional transverse plane sequences in order to keep total imaging time to under 90 minutes.

T2 Weighted Turbo Spin Echo

Sagittal plane

TE 87 TR 5000 Flip Angle 150

No av. 7

Phase encoding right to left

2mm slice thickness

Oblique dorsal plane (perpendicular to long axis of hippocampus)

TE 87 TR 7940 Flip Angle 150

No av. 4

Phase encoding ventral to dorsal

2mm slice thickness

T2 FLAIR

Oblique dorsal plane (perpendicular to long axis of hippocampus)

TE 83 TR 480 TI 1500

No av. 3

Phase encoding ventral to dorsal

3mm slice thickness

T1W 3D MPRAGE

Oblique dorsal plane (perpendicular to long axis of hippocampus)

TE 2.2 TR 1900 TI 500 to 600

No av. 2

isotropic voxels 0.9 mm^3^

SWI (susceptibility weighted imaging - haemorrhage sensitive sequence)

Oblique dorsal plane (perpendicular to long axis of hippocampus)

TE 12.6 TR 20 Flip Angle 9

No av. 1

Phase encoding ventral to dorsal

1.2mm slice thickness
